# Relationships between Stress Granules, Oxidative Stress, and Neurodegenerative Diseases

**DOI:** 10.1155/2017/1809592

**Published:** 2017-01-18

**Authors:** Lihua Chen, Beidong Liu

**Affiliations:** Department of Chemistry and Molecular Biology, University of Gothenburg, P.O. Box 462, Medicinaregatan 9E, S-413 90 Gothenburg, Sweden

## Abstract

Cytoplasmic stress granules (SGs) are critical for facilitating stress responses and for preventing the accumulation of misfolded proteins. SGs, however, have been linked to the pathogenesis of neurodegenerative diseases, in part because SGs share many components with neuronal granules. Oxidative stress is one of the conditions that induce SG formation. SGs regulate redox levels, and SG formation in turn is differently regulated by various types of oxidative stress. These associations and other evidences suggest that SG formation contributes to the development of neurodegenerative diseases. In this paper, we review the regulation of SG formation/assembly and discuss the interactions between oxidative stress and SG formation. We then discuss the links between SGs and neurodegenerative diseases and the current therapeutic approaches for neurodegenerative diseases that target SGs.

## 1. Introduction

Environmental stress can trigger the formation of SGs, including nuclear SGs, which contain heat-shock transcription factor 1/2 (HSF1/2) and pre-mRNA processing factors [1, 2] and also cytoplasmic SGs, which are composed of proteins and mRNAs [3]. This article focuses on cytoplasmic SGs, and the term SG refers to cytoplasmic stress granules hereafter. SGs are transiently formed under stress conditions to reprogram RNA translation by affecting mRNA function and localization and are not associated with other organelles in the cell [4, 5]. SGs typically contain nontranslating mRNAs, translation initiation components such as eukaryotic initiation factor 4G (eIF4G or TIF4631/TIF4632), and additional proteins affecting mRNA function including RNA-binding proteins (RBPs) and non-RNA-binding proteins [6–9]. SGs can be induced by glucose starvation, heat stress, osmotic stress, and oxidative stress, and the composition of SGs can vary depending on the stress [8, 10]. Studies suggest that SG composition varies under different stress conditions and that SGs can form as a consequence of different physical interactions (reviewed in [11]). For instance, SGs induced by glucose deprivation contain eukaryotic initiation factor eIF4E and eIF4G proteins, mRNAs, and the poly(A)-binding protein Pab1 [12, 13], whereas SGs induced by oxidative stress have distinct major components such as eIF2 and downstream factors [6]. In yeast, Gtr1 is essential for SG formation under glucose depletion but suppresses SG formation during heat stress [14]. In both yeast and mammalian cells, Pbp1/Atx2 or Pub1/TIA1 proteins promote SG assembly but are not essential for SG assembly [13, 15]. Moreover, recent evidence indicates that SGs sequester not only transcripts and translation components but also signaling and catalytic proteins. Pathogenic proteins such as fused in sarcoma (FUS1), transactive response DNA-binding protein 43 (TDP-43), and Ras-GTPase-activating protein SH3 domain-binding protein 1 (G3BP1) are recruited into SGs [4, 16–18]. Furthermore, SGs share many components with neuronal granules [19], and mutations that increase SGs are found to be causative in some neurodegenerative diseases (NDs) [20]. SG formation has therefore been closely linked with aging-related diseases such as NDs, which are characterized by continual presence of oxidative stress [21].

Oxidative stress is a well-known SG inducer even though its effects are still controversial. Oxidative stress is caused by imbalanced redox states, owing to either excessive production of reactive oxygen species (ROS) or disturbance of the antioxidant system. Oxidative stress can lead to the damage of cell membranes and other functional components such as proteins, lipids, and DNA. The brain is especially susceptible to these damaging effects because of its high demand for oxygen, its abundance of highly peroxidisable substrates, and its low antioxidant activity (reviewed in [22–24]). Thus, excessive ROS is believed to be a cause of NDs such as Parkinson's disease (PD), Alzheimer's disease (AD), and amyotrophic lateral sclerosis (ALS).

## 2. SG Formation and Oxidative Stress

### 2.1. Regulation of SG Assembly and Disassembly

According to a recently proposed model, SG assembly is based on a liquid-liquid phase separation by the RBPs harboring low-complexity sequence domains [25]. Assembly is initiated by nontranslating mRNP nucleation, which forms an early stable core containing a diverse proteome and a dense network of protein-protein interactions [9]. These cores grow rapidly and are then surrounded by phase-separated shells. Subsequently, the biphasic SGs begin to fuse and form a larger, higher order, mature assembly [26]. Furthermore, recent findings have suggested that SG formation is seeded by aggregation-prone proteins under specific stresses [11, 27–29]. SGs in yeast cells under glucose starvation, for example, tend to form after and on PBs [13]. In mammalian cells, some SGs appear to grow out of preexisting PBs [13, 30]. Together, these reports suggest that SG formation may be initiated through transitions in mRNP composition that occur at PBs. The yeast prion-like protein Lsm4, also a component of the Lsm1-7-Pat1 complex and PBs, has been shown to function as a seed/scaffold for SG formation under certain stress conditions [28].

SGs are dynamic, membraneless organelles that undergo fusion, fission, and flow in the cytosol [31]. They are not uniform in structure and consist of an inner core and a surrounding shell; the core contains higher concentration of proteins and mRNA than the shell, while the shell has lower concentrations of proteins and mRNA but is potentially more dynamic than the core (reviewed in [11]). SGs constantly exchange components with the cytoplasm. Components of SGs have short residence time (seconds) whereas SGs themselves persist for minutes to hours, fusing with each other and with other RNA granules [32]. While the shells of SGs are considered to be relatively dynamic, the cores are thought to be relatively stable. SGs contain a diverse proteome with a dense network of protein-protein interactions in both yeast and mammalian cells [9]. For example, about 50% of SG components within the stable cores are RBPs, and the other non-RBPs are presumably recruited to SGs through protein-protein interactions [9]. Importantly, the identified yeast mRNP proteins and proteome of SGs cores are highly conserved between yeast and mammals [9, 33]. Moreover, many SG proteins harbor prion-like domains that enable the proteins to form self-templating amyloid fibrils [34]. Similarly, low-complexity regions (LCRs), which cause the RBPs to be prone to aggregation in vitro [35], are highly enriched in the proteins essential for SG formation in yeast [14].

Recent proteomic analysis of SG cores revealed that SG assembly is controlled by multiple ATP-driven machineries [9] including (1) ATP-driven disaggregases, (2) DEAD-box helicases, (3) the ATP-dependent VCP-autophagy pathway, (4) processing-bodies (PBs), and (5) other transcription factors. These are discussed in the following paragraphs.

1. * ATP-driven disaggregases*: the yeast Hsp104 disaggregase is an essential protein that dissolves SGs and thereby maintains the integrity of other mRNPs such as PBs by preventing their entry into SGs [27, 28]. Two yeast Hsp110 disaggregases (SSE1 and SSE2) also help dissolve SGs but to a lesser degree than Hsp104 [28]. In contrast, due to the lack of an Hsp104 homolog, mammalian SGs are more liquid-like than their yeast counterparts [28]. Protein disaggregation in mammals is controlled by the Hsp40, Hsp70, and Hsp110 system [36, 37].
2. * DEAD-box helicases*: the unique motifs of DEAD-box helicases are responsible for their important activities, such as ATP binding, ATP hydrolysis, and RNA binding [38]. DEAD-box RNA helicases include yeast Dhh1 and Ded1, human Rck and DDX3,* Drosophila* Me31B,* Plasmodium* DOZI,* Toxoplasma* TgHoDI and TgeIF4a, and* Caenorhabditis* CGH-1, all of which are found in both SGs and PBs [39]. Defects in the ATPase domain of Ded1 or overexpression of* DED1* lead to the accumulation of SGs because Ded1 functions both as a repressor of translation (by forming an eIF4F-Ded1-mRNA complex via interaction with eIF4G) and as an activator of translation through its ATP hydrolysis activity [40]. Upon stress,* DDX3* functions as an SG-nucleator by interacting with its binding partners eIF4E and PABP1 [41]. These examples suggest that DEAD-box helicases are closely linked to SG assembly.
3. * ATP-dependent VCP-autophagy pathway*: a recent screening study revealed that SGs can be eliminated by autophagy. SGs can be targeted to vacuoles by the autophagic process in yeast, and this process is conserved in eukaryotes including mammals [5].
4. * PBs*: PBs affect the assembly of SGs because PBs and SGs are spatially linked and constantly exchange mRNPs [4, 10, 13, 39]. However, SGs can be clearly distinguished from PBs based on morphology/substructure [42] and components [39]. PBs and SGs can dock and/or overlap in both yeast and mammalian cells [13, 31, 42, 43], suggesting that they are dynamically linked sites of mRNPs remodeling.
5. * Other transcription factors*: certain transcription factors that regulate the expression or translocation of some key SG components or mRNA can modulate SG assembly under hostile conditions. In human cells, it is reported that Y-box binding protein 1 (YB-1) directly binds to and translationally activate the 5′ untranslated region (UTR) of* G3BP1* mRNAs, thereby controlling the availability of the G3BP1 SG-nucleator for SG assembly. During oxidative stress, YB-1 is highly activated, which increases SG formation. Inactivation of YB-1 impairs SG assembly and sensitizes cells to oxidative stress [44]. Lyons et al. have recently argued that YB-1 regulates SG formation through a pathway that is independent of G3BP1. YB-1 can bind to tiRNA via its cold shock domain to package the tiRNA-repressed mRNAs into SGs, and this process is dispensable for tiRNA-mediated translational repression [45]. Another important complex that modulates SG assembly is the 40S-G3BP-Caprin-USP10 axis. G3BP associates with the 40S ribosomal subunit via its RGG motif to promote SG assembly, and the G3BP activity is modulated by Caprin and USP10, which can mutually bind to the G3BP protein [46].

### 2.2. Oxidative Stress and SG Formation

#### 2.2.1. The Effect of Oxidative Stress on SG Formation

Oxidative stress has been demonstrated to be an inducer of SG formation. Glucose deprivation induces ROS production [47, 48], which in turn triggers SG formation in yeast cells [13, 14, 49]. In mammals, other oxidative stress reagents including sodium arsenite [26, 50, 51] and hydrogen peroxide [52–55] strongly induce SG formation, although the latter triggers a noncanonical type of SG. Relative to arsenite-induced SGs, hydrogen peroxide-induced SGs are smaller and disassemble more rapidly. These and other structural differences among SGs are probably due to the fact that SG constituents and SG formation processes differ depending on stress conditions. Arsenite-induced SGs, for example, require and contain eIF4E while hydrogen peroxide-induced SGs contain significantly reduced amounts of eIF3, eIF4E, and eIF4G. Moreover, arsenite-induced SGs but not hydrogen peroxide-induced SGs require phosphorylation of eukaryotic translation initiation factor 2 (eIF2) [52]. Although other toxic metals, including methylmercury, lead, and cadmium, also induce oxidative stress and affect the central nervous system [56, 57], there is no evidence that they affect SG formation. Arsenite-induced oxidative stress is known to promote transfer RNA (tRNA) cleavage and accumulation of tRNA-derived small RNAs in an angiogenin- (ANG-) dependent manner [58]. These tiRNAs could inhibit translation initiation and induce the assembly of SGs by binding to YB-1. Thus, the secreted ribonuclease ANG is linked with the oxidative stress response and SG assembly [45, 58, 59]. Multiple mutations of ANG have been identified in ALS and Parkinson's disease patients [60–62], probably because the protein is highly active in motor neurons and other components of the central nervous system. Structural and molecular analysis of the pathological human ANG mutations has revealed that the structure of mutated ANGs is correlated with their effects on SG assembly in neuronal cell lines [63].

Besides of the SG formation-inducing effects, oxidative stress might also suppress SG formation. Thedieck et al. reported that ROS such as hydrogen peroxide oxidize the SG-nucleating protein TIA-1, thereby inhibiting SG assembly [64]. Moreover, hydrogen peroxide also attenuates arsenite/ER stress-induced SG formation, although a high concentration of hydrogen peroxide (1 mM) slightly induces SG formation. Researchers have inferred that hydrogen peroxide is able to directly oxidize the key SG-nucleating protein TIA-1 and thus suppresses SG assembly by impeding the interaction between TIA-1 and its target mRNAs [15, 64].

#### 2.2.2. The Effect of SG Formation on Oxidative Stress Defense

Emerging evidence indicates that SG formation plays an active regulatory role in the response of cells to oxidative stress. SGs have antioxidant activity that is controlled by two SG components, the antioxidant enzyme USP10 and its cofactor G3BP1; G3BP1 can mask USP10 activity under steady-state conditions. Under oxidative stress such as sodium arsenite treatment, SGs form to inactivate the G3BP1 and to thus activate USP10, which results in a decrease in ROS production and apoptosis. Under H_2_O_2_ stress, no SGs form, and the cells are more prone to apoptosis [65]. USP10 interacts with many proteins localized at polysomes, such as PABPs, HuR, RACK1, and YBX1 [66]. Therefore, USP10 might control the stability and/or translation of mRNA(s) involved in redox control.

## 3. SGs and NDs

### 3.1. Links between SGs and NDs

**Figure 1:**
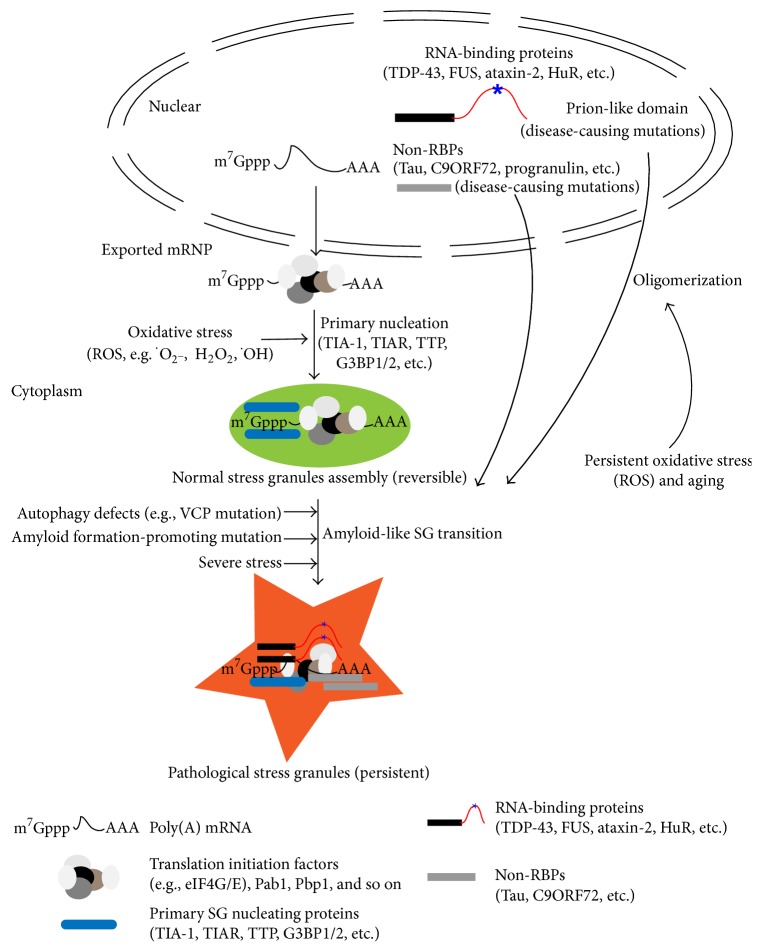
Schematic diagram of normal stress granule (SG) formation and the transition from normal SGs to pathological SGs. During transient oxidative stress such as ROS, translation of mRNA is stalled, and the nuclear exported nontranslating mRNPs (mRNA and many translation initiation factors such as eIF4G/E, Pab1, and Pbp1) form normal SGs in the cytoplasm through sequestration of RNA-binding proteins like primary nucleators (G3BP1/2, TIA-1, and TIAR). These SGs are reversible and dynamic, and they exchange components with the cytoplasm. Severe stress or mutations that decrease SG clearance or that enhance amyloid-like aggregation or can cause normal SGs to become pathological, irreversible SGs. Moreover, mutations in many RNA-binding proteins (TDP-43, FUS, ataxin-2, HuR, etc.) and non-RNA-binding proteins (tau, C9ORF72, etc.) can accelerate this transition via their self-aggregation (oligomerization), which is promoted by persistent oxidative stress or aging.

The close interaction between SGs and the insoluble protein aggregates that accumulate in NDs clearly indicates that SGs affect NDs. Studies have suggested a model for the possible transition from SGs with normal dynamics to those that are pathological. Normal cytoplasmic SGs assemble in response to various stressful conditions via the intrinsic prion-like domains. These SGs can be well managed in the normal dynamic (e.g., their assembly can be reversed once the stress is absent), or they can form super-stable amyloid-like assemblies under the following conditions: (1) the stress becomes more severe (e.g., heat shock [28]); (2) mutations occur that promote SG assembly (e.g., prion-like domains in hnRNPs [67]) or amyloid formation; or (3) mutations occur that limit clearance (e.g., VCP/CDC48) (reviewed in [21, 68]) (Figure 1). The persistent mRNP granules could impair ribostasis and cause other pathological changes in the cells. Although SGs are not neuron- or glia-specific and are present and active in most types of cells, they have disproportionate effects on neurons and muscle cells. This is probably because of the longevity of these cells (which enables age-related damage to accumulate to pathological levels) and because of their unique architecture and connectivity (reviewed in [21]). For instance, neurons contain SGs that are composed of components required for synaptic plasticity [69] and that are related to neuronal RNA transport for spatial control of local protein translation [70].

NDs are often characterized by pathological inclusions, a subset of which colocalizes with SG markers. Many RBPs, especially the primary nucleating proteins including T-cell-restricted intracellular antigen-1 (TIA-1), TIA-like-1 (TIAR), tristetraprolin (TTP), and G3BP1/2, have been characterized as classic SG markers and have been found to be associated with pathological lesions in neurodegeneration diseases [6, 71, 72]. SGs can be visualized by in situ hybridization with oligo-dT probes against the polyadenylated mRNAs that are trapped in SGs [6, 71] or by immunofluorescent approaches that detect classic SG marker proteins like TIA-1, G3BP1, and PABP [73, 74]. In ALS and FTLD-TDP, SGs can be characterized by detecting TDP43-positive lesions [75]; in the AD and frontotemporal dementia, SGs can be detected by detecting tau lesions with tau (FTLD-tau) [76]. SGs are structurally different from amyloidogenic deposits, which are fibrillar aggregates that are also associated with NDs. Dementia with Lewy bodies caused by alpha-synuclein fibrils, for example, is not associated with SGs (reviewed in [68]). In some ALS and FTLD cases, however, the pathological SGs are colocalized with amyloidogenic deposits because the SG marker proteins TIA-1 and TDP-43 can form both SGs and insoluble fibrillary aggregates under pathological conditions and because SGs may function as seeds for irreversible aggregation (reviewed in [77]). Moreover, SGs contain many ubiquitin-modified proteins such as HADC6 [78]. Therefore, in some NDs that are SG-positive, ubiquitinated aggregates of SG marker proteins like TDP-43 and TIA-1 can be observed [79, 80].

In diseased brain tissues and cultured cells, mutations of many RBPs such as TDP-43, FUS, ataxin-2, the survival motor neuron (SMN), optineurin (OPT), and ANG colocalize with core SG markers [68, 75, 81, 82]. However, in some neurological disorders associated with SG-positive pathology, the SG-related bioprocess is associated with mutations in other non-RBPs factors like progranulin (PGRN) [83] and C9ORF72 [84]. Null mutations in PGRN lead to some cases of FTLD-U [85, 86]. In addition, TDP-43 protein accumulates in FTLD caused by the loss-of-function of PGRN; this indicates a tight connection of PGRN to SG assembly and NDs because TDP-43 pathology has been observed in a spectrum of NDs, including FTLD-U, ALS, ALS–FTLD, AD, and Guam Parkinson dementia complex (reviewed in [83]). The GGGGCC (G4C2) intronic repeat expansion in the C9ORF72 gene is a common genetic cause of familial ALS and frontotemporal dementia. This loss-of-function mutation leads to reduced axonal actin dynamics via interaction with cofilin, which may contribute to NDs such as ALS and FTD when combined with toxicity like dysregulated RNA metabolism [87]. In addition, the SG marker proteins (e.g., TIA1 and TTP) also colocalize and interact with the aggregated phosphorylated tau in AD and in FTLD-tau [76]. The pathological SGs formed from TIA-1, TTP, and G3BP are less insoluble than the pathological aggregates of tau or *β*-amyloid, while the TDP-43 and FUS can form highly insoluble inclusions [75, 76, 81]. All of this evidence indicates that SG proteins participate in neurodegeneration diseases by interacting with the pathological aggregates of RBPs or non-RNPs and that SGs may be involved in the pathology of a broad spectrum of neurodegenerative disorders (Figure 1).

As one of the most metabolically active regions in the body, the brain is especially vulnerable to oxidative stress. Oxidative stress can lead to the oxidation of proteins and, in turn, to changes in tertiary structure that promote protein aggregation [88, 89]. Moreover, early stage protein aggregation generates hydrogen peroxide and other ROS, suggesting that there may be a common, fundamental molecular mechanism underlying the pathogenesis of oxidative damage and neurodegenerations [90, 91]. Acute oxidative stress also promotes SG formation, and persistent oxidative stress facilitates the oligomerization of pathological RBPs or non-RBPs such as TDP43, FUS, tau, and C9ORF72. The oligomers are subsequently sequestered into SGs to further enhance the transition to pathological amyloid-like SGs (Figure 1). It is unclear whether hyperactive SG formation is beneficial or harmful to the cell. SGs that form in response to acute stress are protective and antiapoptotic. In aging-associated diseases like neurodegeneration, however, the stress is chronic and cannot be resolved. The sustained, overactive SGs in neurons may interfere with neuronal function by silencing transcripts and by sequestering important proteins such as RNPs.

### 3.2. ND Therapies That Target SGs

Based on the close relationships between SGs, oxidative stress, and NDs, researchers have proposed the following four novel approaches to reverse pathological SGs and to perhaps delay the progression of diseases [68, 76]:

1. Intervene in eIF2*α* phosphorylation. The discovery of overactive SG formation in other diseases raises the possibility that the underlying pathways are overactive in multiple NDs and other aging processes and that pharmacotherapy targeting SG formation might be protective. Because SG formation induced by oxidative stress mainly depends on eIF2*α* phosphorylation signaling and because phosphorylated eIF2*α* is elevated in sporadic AD brains [92], SG assembly might be inhibited by reducing eIF2*α* phosphorylation. In addition, aggregated phosphorylated tau in AD is also generated by the eIF2*α* pathway (PERK and PKR) via activation of a major tau kinase in the brain, glycogen synthase kinase-3*β* [93, 94]. A recent study tested the possibility of treating ND by targeting eIF2. The study, which used an animal model of Creutzfeld-Jakob disease in which pathological misfolding of PrP precipitates neurodegeneration, revealed that a reduction in eIF2*α* phosphorylation reduced PrP-induced neurodegeneration and that an increase in eIF2*α* phosphorylation increased SG formation and accelerated neurodegeneration [95]. Physical activity that prevents activation of eIF2*α* phosphorylation is able to delay AD progression [96]. These results suggest that targeting the SG pathway by inhibiting eIF2*α* phosphorylation can inhibit neurodegeneration.
2. Target major SG components like TDP-43. Neurodegeneration mediated by TDP-43 is linked to complex pleiotropic effects of protein translation dysregulation and SG biology. Therefore, targeting TDP-43 pathophysiology to reduce TDP-43 aggregation may be effective to inhibit neurodegeneration [97]. Researchers have identified FDA-approved chemicals that moderately reduce TDP-43 aggregation [98] and have developed a series of novel compounds that strongly reduce TDP-43 aggregation with minimal toxicity [99].
3. Target factors regulating SG assembly. An extensive body of evidence demonstrates that histone deacetylase 6 (HDAC6) is involved in NDs such as HD [100] and PD [101]. HDAC6 is also essential for SG assembly because it mediates the motor-protein-driven movement of individual SG components along microtubules [78]. Moreover, HDAC6 interacts with p97/VCP, an AAATPase (an ATPase associated with a variety of activities) that is directly involved in protein degradation [102] and SG clearance [5]. Therefore, selective HDAC inhibitors [103, 104] are being studied because they are able to target multiple signaling pathways including SG formation, oxidative stress accumulation [105], and protein aggregation. Some newly developed HDAC6 inhibitors such as tubacin and tubastatin A have been evaluated as potential agents for treating NDs like AD [106], PD [107], HD [100], and others [108, 109].
4. Target the oxidative stress defense system. It remains unclear whether oxidative stress is the cause or consequence of NDs [110]. Exploratory reports and clinical data have thus far indicated that oxidative stress is a ubiquitously observed hallmark of neurodegenerative disorders [111, 112]. In this regard, approaches focusing on redox signaling and related antioxidant enzymes may be able to delay the diseases [112, 113]. In PD and AD, the strongest alteration in the antioxidant defense is a decrease in GSH concentration [114–116]. Medicinal chemistry-based strategies to increase GSH levels, including the use of analogues as well as prodrugs and codrugs, have been well assessed in vivo and in vitro (reviewed in [113]). Other strategies to enhance the redox system include the stimulation of Nrf2 (the master protein that regulates the redox homeostasis) and the reduction in ROS by medical gases like carbon monoxide, hydrogen sulfide, and hydrogen (reviewed in [112]). However, whether these modifications of the redox system affect SG formation/function remains to be determined.

## 4. Perspectives

NDs are complicated and cannot be attributed to a single gene or even multiple genes. They are caused by unknown signaling cascades, misfolded proteins, ubiquitin-proteasome dysfunction, oxidative stress, and many other events. Increasing evidence suggests that the formation of RNA granules and especially of SGs is central to many NDs. The physiological aggregation of RBPs becomes pathological when the proaggregation state is favored because of mutations or oxidative stress. The mutation of SG-related RBPs may shift the equilibrium of these RBPs and lead to increased SG formation and the formation of the stable, long-lived protein aggregates that are associated with disease pathology [117, 118]. This alteration could create conditions that enhance subsequent disease development. The increased aggregates may also function to facilitate the formation of secondary mature SGs around the aggregates [119], which could lead to excessive SG formation.

Despite recent advances concerning our understanding of SG biology and NDs, two major questions related to SG biology remain to be answered. First, what are the consequences of SG persistence? Sustained SGs might protect the neuron by facilitating the cellular response to oxidative stress or by sequestering toxic oligomers. However, SGs might also recruit RBPs that interfere with their normal function. Second, does crosstalk between SG formation and the ubiquitin-proteasome system (UPS) affect the pathophysiology of NDs? We know that some UPS components like USP10 also participate in SG assembly and that both UPS components and SGs are implicated in the pathogenesis of NDs. Determining how the two processes interact and affect neurodegeneration should suggest new ways to treat NDs.
